# Is there a difference in FDG PET findings of invasive ductal carcinoma of the breast with and without coexisting DCIS?

**DOI:** 10.22038/aojnmb.2019.41658.1284

**Published:** 2020

**Authors:** Ismet Sarikaya, Ali Sarikaya, Ahmed N. Albatineh, Ebru Tastekin, Yavuz Atakan Sezer

**Affiliations:** 1 Faculty of Medicine, Department of Nuclear Medicine, Kuwait University and Mubarak Al-Kabeer Hospital, Kuwait; 2 Faculty of Medicine, Department of Nuclear Medicine, Trakya University, Edirne, Turkey; 3 Faculty of Medicine, Department of Community Medicine and Behavioral Sciences, Kuwait University, Kuwait; 4 Faculty of Medicine, Department of Pathology, Trakya University, Edirne, Turkey; 5 Faculty of Medicine, Department of Surgery, Trakya University, Edirne, Turkey

**Keywords:** FDG PET, Breast carcinoma, Invasive ductal carcinoma, Coexisting DCIS

## Abstract

**Objective(s):**

Studies have reported that invasive ductal carcinoma (IDC) with coexisting ductal carcinoma in situ (DCIS) show lower metastatic potential and recurrence and better overall survival than pure IDC. In this study, we assessed F-18 fluorodeoxyglucose (FDG) positron emission tomography/computed tomography (PET/CT) images of patients with newly diagnosed IDC to determine if there is any difference in PET findings in IDC-DCIS and pure IDC cases.

**Methods:**

FDG PET/CT images of patients with newly diagnosed IDC of the breast who subsequently underwent breast surgery and had histopathology result in our records were further evaluated. Tumor grade, pathological staging, and presence of DCIS were noted from the histopathology results. Standardized uptake value (SUV) of the primary tumor (SUV_max_ and SUL_max_), other hypermetabolic foci in the breast, and ipsilateral normal breast were measured. Presence of axillary and distant metastases was noted.

**Results:**

Fifty seven (57) patients with IDC were included. Coexisting DCIS was present in 44 (IDC-DCIS) and not present in 13 (pure IDC) cases. Per histopathology, the primary tumor was unifocal in 33 IDC-DCIS (75%) and 12 pure IDC (92.3%) cases, and multifocal in 11 IDC-DCIS cases (25%), and 1 pure IDC case (7.7%). FDG uptake was multifocal in 20 IDC-DCIS cases (45.5%) and 1 pure IDC case (7.7%), and unifocal in 24 IDC-DCIS (54.5%), and 12 pure IDC (92.3%) cases. There was no significant difference in patient age, size of the primary tumor, SUV_max_ and SUL_max_ of the primary tumor and SUV_max_ of the normal breast in IDC-DCIS and pure IDC cases (p>0.05). Pathology showed axillary metastasis in all 13 pure IDC (100%), and 27 IDC-DCIS (61.4%) cases. PET showed axillary uptake in 25 IDC-DCIS (56.8%), and 8 pure IDC (61.5%) cases, and abnormal/questionable distant uptake in 12 IDC-DCIS cases and 1 pure IDC case.

**Conclusion:**

In our preliminary findings, multifocal breast FDG uptake and multifocal tumor appear to be more common in IDC-DCIS than pure IDC. There is no significant difference in SUV and size of the primary tumor in IDC-DCIS and pure IDC cases. Axillary metastases appear to be more common in pure IDC than IDC-DCIS cases.

## Introduction

F-18 fluorodeoxyglucose (FDG) positron emission tomography/computed tomography (PET/CT) is commonly used in the detection of distant metastases in high-risk patients with locally advanced disease or inflammatory breast cancer (1, 2). FDG PET is most helpful in situations where standard staging studies are equivocal or suspicious especially in the setting of locally advanced or metastatic disease (3). Studies have also reported that staging with FDG PET/CT might be of value in intermediate-risk patients (4, 5).

 Ductal carcinoma in situ (DCIS) is a preinvasive/non-invasive breast cancer, where proliferations of malignant ductal epithelial cells remain confined within intact breast ducts. DCIS often coexist with invasive ductal carcinoma (IDC) (6). DCIS is recognized as a precursor of IDC although some proposed that it was not the precursor (6-9). DCIS has the potential to transform into an invasive cancer over time. In tumors lacking DCIS, it is assumed that IDC arises de novo (6, 7). Various studies with controversial results have been performed to compare IDC-DCIS to pure IDC (6, 7, 10-14). Wong et al. reported that IDC with coexisting DCIS was characterized by lower proliferation and metastatic potential than size-matched pure IDC, especially if the ratio of DCIS to IDC size was high (7). Compared with pure IDC, IDC-DCIS tumors were more often positive for estrogen receptor (ER), progesterone receptor (PR) and human epidermal growth factor receptor/HER2, and had lower grade and Ki-67 (7). In another study, Wong et al. have concluded that the presence of coexisting DCIS in IDC predicts lower biological aggressiveness in luminal cancers but not in the conventionally more aggressive HER2-positive and triple-negative subtypes (10). Dieterich et al. reported that IDC accompanied by DCIS was associated with lower local recurrence (6). Patients with IDC-DCIS were significantly younger, had smaller tumors, and less lymph node involvement and local recurrence rate was significantly increased in patients with pure IDC (6). Presence of DCIS was associated with a trend towards superior disease-free survival and overall survival, but it was not an independent predictor of improved outcome (11). Lower expression of HER2/neu and Ki67 were found in IDC-DCIS patients as compared to pure IDC patients (12). Breast density, Tc-99m V-dimercaptosuccinic acid (Tc-99m V-DMSA) uptake, calcitonin gene related peptide, and Ki-67 were significantly increased, whereas ER was significantly decreased in IDC-DCIS as compared to pure IDC (13). Merchera et al. reported that concomitant DCIS was one of the significant predictors of tumor recurrence by univariate analysis (14).

In this study we aimed to further assess F-18

FDG PET images of newly diagnosed IDC patients to determine if there is any difference in PET findings in IDC-DCIS and pure IDC cases.

## Methods

 In this study, FDG PET/CT images of newly diagnosed and untreated breast cancer patients who subsequently underwent breast surgery and had histopathology result in our records were further evaluated. This retrospective study was approved by Kuwait Ministry of Health and Health and Ethics Committee at Trakya University Faculty of Medicine. 

 FDG PET/CT images were obtained at Philips Gemini Time of Flight (Philips Medical Systems, Best, Netherlands) and GE discovery 8 (General Electric Medical Systems, Milwaukee, WI, USA) PET/CT cameras. PET images were obtained 60 min following intravenous injection of 222-296 MBq (6-8 mCi) of F-18 FDG. Prior to PET image acquisition, a low dose CT was obtained for attenuation correction and anatomic localization purposes. PET acquisition was 3 min/bed from top of the head to mid thighs. PET images were corrected for attenuation on the basis of the CT data and reconstructed using a standard iterative algorithm (ordered subset expectation maximization) and reformatted into transaxial, coronal and sagittal views. Maximum intensity projection images were also generated. Both attenuation corrected and uncorrected PET images as well as PET/CT fusion images were reviewed. 

 FDG PET/CT images were evaluated by 2 board certified Nuclear Medicine physicians. We assessed ipsilateral breast for the primary tumor and other foci of uptake in the breast and also distribution of activity in both breasts and viewed the images for presence of nodal and distant metastases. We measured maximum standardized uptake values (SUV_max_) of the primary tumor(s), focal hypermetabolic areas other than the primary tumor (multifocal uptake), and ipsilateral normal appearing breast tissues by placing circular region of interests. We also normalized SUV_max_ to lean body mass (SUL_max_) in primary tumors. We calculated patient’s lean body mass via an online calculator which uses an equation using patients’ height and weight (15). 

 Location of the multifocal FDG uptake was noted as adjacent to, around (within 1-2 cm) and away from primary tumor. Histopathology results were reviewed mainly to obtain information about pathological stage of the tumor, grade of the primary tumor, presence of multifocal tumor, and presence of DCIS. In patients with partial surgery, such as wide local excision, lumpectomy, or segmental mastectomy, DCIS adjacent/around the tumor was searched pathologically. 

***Statistical Analysis***

 Statistical analysis was performed using SPSS software version 25 (IBM Corp.). The goal was to determine if there was any significant difference in mean SUV_max_, SUL_max_ and mean size of the primary tumor, mean SUV_max_ of multifocal uptake and mean SUV_max_ of the ipsilateral normal breast in IDC-DCIS and pure IDC cases. To compare means and calculate p values, the two-sample t test was used if normality holds or the non-parametric Mann–Whitney U test for non-normal data. P< 0.05 was considered statistically significant.

## Results

 Fifty seven (57) female patients with newly diagnosed IDC from Mubarak Al-Kabeer (29 patients) and Trakya University (28 patients) hospitals were selected for further analysis (mean age 57.1±12.2 year). 

Thirty four (34) patients had underwent mastectomy (9 modified radical), 14 had breast preservation surgery (lumpectomy or segmental mastectomy), and 9 had wide local excision. All the patients had undergone sentinel lymph node (SLN) biopsy and/or axillary clearance/ dissection. 

Table 1 shows pathological stage, grade of the primary tumor, presence of DCIS and SUV_max_ and SUL_max_ of primary tumor. 

Coexisting DCIS was present in 44 patients with IDC (IDC-DCIS). There was no coexisting DCIS in 13 patients (pure IDC). Histopathologically, the primary tumor was multifocal in 12 patients (in 11 IDC-DCIS cases and in 1 pure IDC case) and unifocal in 45 patients (in 33 IDC-DCIS, and 12 pure IDC cases). 

**Table 1:** Pathological stage, grade, presence of DCIS, SUV_max_ and SUL_max_ of primary tumor in patients with IDC of the breast

**Pathological Stage**	**Grade**	**DCIS**	** SUV**_max_	** SUL**_max_
T2N0(SN)	3	+	12.3	7.4	
T2N0(SN)	3	+	11.7	7.1	
T1CN1A	2	+	3.9	2.2	
T2N2A	3	+	9.3	6.5	
T2N1A	2	+	8.7	4.4	
T2N0(SN)	2	+	2.4	1.4	
T2N0	2	+	3.7	2.2	
T2N0(SN)	3	+	7	4	
T1AN0(SN)	2	+	3.8	1.9	
T2N0(SN)	1	+	8.3	5.3	
T2N0(SN)	2	+	3.2	1.8	
T3N2A	2	+	7.4	4.3	
T3N0	2	+	5.8	3.2	
T2N2A	3	+	12	7.6	
T3N2A	3	+	5.7	3.7	
T2N2A	3	+	9.2	5.1	
T2N3A	2	+	8.9	4.8	
T2N2A	3	+	5.4	2.8	
T2N3A	2	+	8.4	5.1	
T2N1A	3	+	5.4	3.3	
T1BN0	1	+	3	1.9	
T2N0	3	+	5.1	2.8	
T1CN2A	2	+	5.7	3.3	
T2N2A	2	+	15.4	9	
T1AN0	1	+	4.4	3.2	
T1BN0	1	+	13	9	
T2N3	2	+	7.6	4.9	
T2N2A	2	+	12	7.2	
T2N3A	2	+	6.5	3.6	
T2N2A	3	+	20	11.8	
T3N3	3	+	8.9	5.8	
T1BN1A	2	+	4.6	2.8	
T1BN2A	3	+	12.4	8.4	
T2N3A	3	+	6.4	3.7	
T3N2A	3	+	8.1	4.8	
T2N1A	2	+	18.8	11.3	
T2N0(SN)	3	+	7.8	4.6	
T2N2A	3	+	6.2	3.4	
T2N3A	2	+	3.3	2.1	
T2N0	3	+	13.4	10.8	
T1CN0(SN)	1	+	2.1	1.4	
T1CN0(SN)	2	+	4	2.6	
T1CN0(SN)	2	+	5	3.1	
T2N1A	2	+	9.6	4.3	
T2N1A	3	-	5.5	2.9	
T2N1A	2	-	8	5.2	
T2N2A	2	-	5.7	3.4	
T2 N1A	2	-	10.1	5.9	
T1CN1(SN)	3	-	5.7	3.4	
T4BN3A	3	-	9.9	6.8	
T1CN2A	3	-	3.5	2.3	
T1CN2A	2	-	5.7	3.6	
T4N1C	3	-	11.3	7.9	
T3N2A	1	-	6	3.7	
T2N2A	3	-	5.5	3.9	
T2N2A	3	-	7.4	4.2	
T1AN1A	1	-	6.1	3.7	

Table 2 summarizes PET (SUV_max_ and SUL_max_ of primary tumor and SUV_max_ of normal breast) and pathology findings of IDC-DCIS and pure IDC patients and statistical results. 

 In IDC-DCIS group (44 patients), FDG PET showed multifocal uptake in 20 patients (45.5%) and unifocal uptake in 24 patients (54.5%). Multifocal FDG uptake was present in 9 of 11 patients with multifocal tumor, and in 11 of 33 patients with unifocal tumor on pathology. 

**Table 2:** FDG PET/CT and pathology findings in IDC-DCIS and pure IDC cases

	**IDC-DCIS**	**Pure IDC**
**Number of patients**	44	13
**Pathology**		
-Unifocal tumor	33	12
-Multifocal tumor	11	1
**PET**		
-Unifocal uptake	24	12
-Multifocal uptake	20	1
**Mean SUV**_max_ of primary tumor*	7.7±4.1	7.0±2.3
**Mean SUL**_max_ of primary tumor**	4.8±2.7	4.4±1.6
**Mean SUV**_max_ of normal breast***	1.2±0.5	1.2±0.6

*p: 0.739, **p: 0.902, ***p: 0.753

In IDC-DCIS group, mean SUV_max_ of multifocal uptake was 4.3±2.3, ranging from 2 to 10.4. Number of multifocal uptake (foci of uptake in addition to primary tumor) was 1 in 10 patients (5 adjacent to primary tumor and 5 away from primary tumor), 2 in 3 patients (adjacent to primary tumor), 3 in 3 patients (some adjacent, some around and some away from primary tumor) and ≥ 5 in 3 patients (some adjacent, some around and some away from primary tumor).

**Figure 1:**
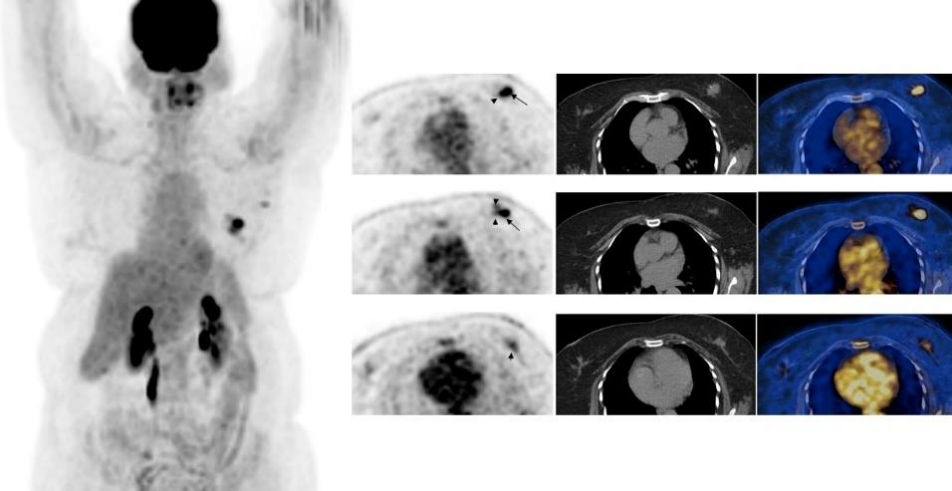
F-18 FDG whole body maximum intensity projection image and selected transaxial PET, CTand PET/CT fusion images of the breasts of a 60 year-old patient with left invasive ductal carcinoma who underwent lumpectomy, sentinel node biopsy and axillary dissection. Histopathology showed unifocal tumor, 4.4×4×2.2 cm in size, primary tumor grade 3, pathological stage T2N1A as well as ductal carcinoma insitu. PET showed the hypermetabolic primary tumor (SUV_max_=5.4) (arrow) and mildly hypermetabolic 3 other foci adjacent/around the primary tumor with SUV_max_ 1.6 to 3.5 (arrow heads) (normal breast SUV_max_=0.8) and mildly hypermetabolic left axillary lymph nodes (SUV_max_=1.9). Mild focal uptake in the right breast could be physiological or not

**Figure 2:**
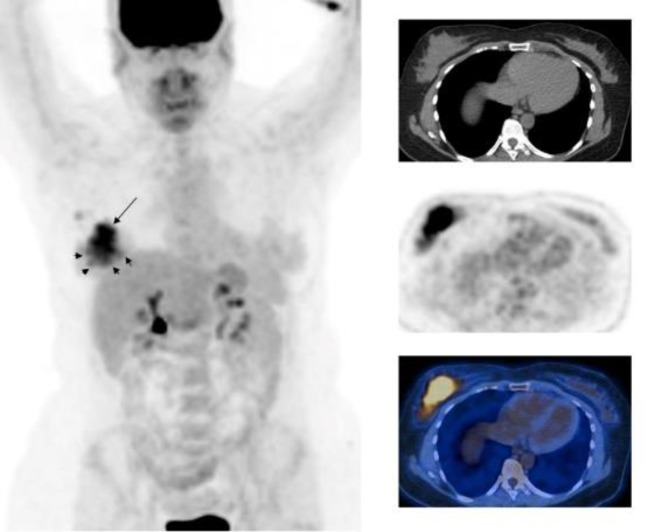
F-18 FDG whole body maximum intensity projection image and selected transaxial CT, PET and PET/CT fusion images of the breasts of a 51 year-old female with right invasive ductal carcinoma who underwent mastectomy, sentinel node biopsy and axillary dissection. Histopathology showed unifocal tumor, 11.3×5.5×3 cm in size, primary tumor grade 2, pathological stage T3N2A as well as ductal carcinoma insitu. PET showed a large hypermetabolic primary tumor (SUV_max_=7.4) (arrow) and multiple small foci surrounding the primary tumor with SUV_max_ around 2.7 (short arrows) and mildly hypermetabolic right axillary lymph nodes (SUV_max_=2.8)

**Figure 3:**
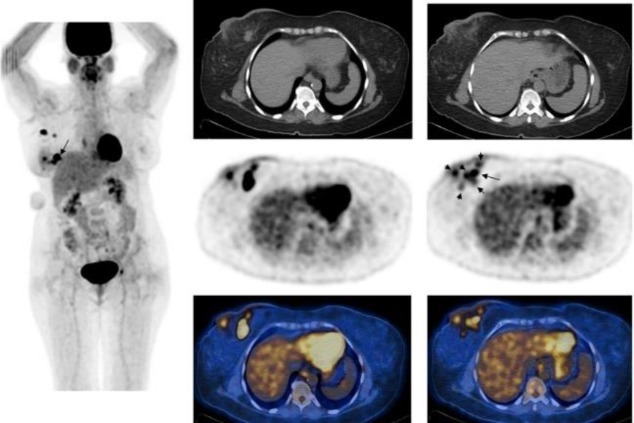
F-18 FDG whole body maximum intensity projection image and selected transaxial CT, PET and PET/CT fusion images of the breasts of a 58 year-old patient with right invasive ductal carcinoma who underwent right mastectomy and axillary dissection. Histopathology showed primary tumor in lower inner quadrant, 2.4×1.7×1.5 cm in size, with retroareolar extension and multiple microscopic foci (primary tumor grade 3, pathological stage T2N2A) as well as ductal carcinoma insitu surrounding the main tumor. PET showed the hypermetabolic primary tumor (SUV_max_=12) (arrow) and multiple smaller hypermetabolic foci surrounding the primary tumor and also in retroareolar region (SUV_max_=3.4, 3.7, 4.1 and 4.4) (short arrows) as well as right axillary lymph node metastases (SUV_max_=8.9). Diffuse uptake is also seen in the skin of the right breast, indicating extension of the tumor

 In IDC-DCIS group, in 7 of 11 patients with unifocal tumor on pathology but multifocal uptake on PET, surgery was partial (lumpectomy, excisional biopsy or breast preserving surgery) and in these patients multiple foci of uptake were around the primary tumor in 4, around and away from the primary tumor in 1 and away from the primary tumor in 2 cases (Figure 1). In 4 of 11 patients with unifocal tumor on pathology but multifocal uptake on PET, surgery was mastectomy and multifocal uptake was around the primary tumor in 3, and around and away from primary tumor in 1 patient (Figure 2). In 2 of 11 patients with multifocal tumor on pathology, PET showed unifocal uptake (primary tumor), no other foci. In one of these patients, size of the multifocal tumor was 4 mm which is below PET resolution. In a patient with microscopic multifocal tumor and mastectomy as surgical procedure, PET showed multiple foci of uptake (at least 5 foci with highest SUV_max_ of 4.4) adjacent, around and away from the primary tumor (SUV_max_ of 12) (Figure 3). 

 In another case with microscopic multifocal tumor and lumpectomy, PET showed uptake in the primary tumor (SUV_max_ of 5.4) and also another focal uptake adjacent to primary tumor (SUV_max_ of 2). In a patient with multifocal tumor on pathology and multifocal breast uptake on PET (primary tumor SUV_max_ of 8.9, multifocal uptake SUV_max_ of 2.7), coexisting lobular carcinoma in situ (LCIS) was also detected in addition to DCIS. In this group, 25 patients had mastectomy and 19 had partial surgery. In patients with partial surgery, DCIS was available around the tumor and other parts of the breast were not examined. 

 In pure IDC group, pathology showed unifocal tumor in 12 patients (92.3%) and multifocal tumor in 1 patient (7.7%). PET showed unifocal uptake in 12 patients (92.3%) and multifocal uptake in 1 patient (7.7%). In a case with unifocal tumor in pathology, PET showed focal uptake (SUV_max_ of 3.9) in addition to primary tumor. There was coexisting LCIS in this patient. In a patient with multifocal tumor on pathology, PET showed uptake in the primary tumor only. In 4 patients in this group, surgery was partial and DCIS was negative around the tumor but whole breast was not examined to search for DCIS in other parts of the breast. 

 Overall, multifocal uptake and multifocal tumor was more common in IDC-DCIS cases than pure IDC. Histopathologically proven, multifocal tumor was present in 25% of IDC-DCIS and 7.7% of pure IDC cases. Multifocal uptake was present in 45.5% of IDC-DCIS and 7.7% of pure IDC cases. 

 Pathology showed axillary lymph node metastasis in all 13 pure IDC cases (100%), and in 27 IDC-DCIS cases (61.4%). PET showed uptake in the axillary nodes in 25 IDC-DCIS patients (56.8%), and in 8 pure IDC patients (61.5%). PET was false negative for axillary metastases in 5 cases with IDC-DCIS and 5 cases with pure IDC. PET was false positive for axillary metastases in 3 cases with IDC-DCIS.

 PET showed findings suggestive of/consistent with distant metastases in 6 IDC-DCIS cases (in bone in 4 cases, in liver in 1 case, and in lung in 1 case), and 1 pure IDC case (in bone). In 6 other cases with IDC-DCIS there was questionable or suspicious uptake: mildly hypermetabolic mediastinal and abdominal lymph nodes (3 cases), mild uptake in lung nodules (2 cases), and questionable uptake in a rib (1 case). In 3 cases with IDC-DCIS there was abnormal uptake in thyroid which was likely due to thyroid pathology. 

***Results of statistical analysis***

 There was no significant difference in mean age of patients in IDC-DCIS (57±12.5) and pure IDC (60.4±10.4) cases (p=0.519).

 There was no significant difference in mean SUV_max_ of the primary tumor in IDC-DCIS (7.7±4.1) and pure IDC (7.0±2.3) cases (p=0.739).

 There was no significant difference in mean SUL_max_ of the primary tumor in IDC-DCIS (4.8±2.7) and pure IDC (4.4±1.6) cases (p=0.902).

 There was no significant difference in mean greatest dimension of the primary tumor in IDC-DCIS (3.3±2.2) and pure IDC (3.9±2.7) cases (p=0.617).

 There was no significant difference in mean ipsilateral normal breast uptake in IDC-DCIS (1.2±0.5) and pure IDC (1.2±0.6) cases (p=0.753).

## Discussion

 Use of FDG PET scan is well established in breast cancer. FDG avidity differs among histological subtypes of the breast cancer. FDG uptake is usually high in IDC and lower in invasive lobular carcinoma (1, 16). FDG uptake in breast cancer is also correlated with tumor grade and tumor cell proliferation (Ki-67 expression) (1, 17). F-18 FDG uptake is negatively correlated with hormonal receptor status. Estrogen and progesterone receptor negative tumors show higher FDG uptake (16, 18). Triple-negative breast cancer (negative for ER, PR and HER2), have poor prognosis and usually show high FDG uptake (18, 19). DCIS usually show low FDG uptake (20). Fujioka et al. reported FDG uptake in 53.8% of DCIS tumors with mean±SD SUV_max_ of 2.18±1.16 (range, 1.16-5.49) (21). In their study, symptomatic and large DCIS (≥ 20 mm) often visualized on FDG PET/CT. In an FDG PET study in DCIS patients, tumor cell density of intraductal carcinoma appears strongly correlated to detection of DCIS by FDG-PET/CT (21). FDG-PET was positive in 8 of 19 patients with DCIS (SUV range of 0.6-2.8) (23). PET was positive only when the size of in situ carcinoma was higher greater than 1 cm (23). FDG uptake was higher in DCIS with microinvasion than pure DCIS (24).

 Coexisting DCIS was reported in 32% and 63.1% of cases with IDC (25, 26). Studies have reported that patients with IDC of the breast who have also coexisting DCIS show lower metastatic potential and recurrence and better overall survival than the patients with pure IDC although there are also some controversial results (6,7,10-14). In the literature, there is only 1 radionuclide study comparing IDC-DCIS to pure IDC (13). With the current study we wanted to determine if there are different findings on FDG PET scan in IDC-DCIS and pure IDC cases. We did not find a significant difference in mean size, mean SUV_max_ and mean SUL_max_ of the primary tumor in IDC-DCIS and pure IDC cases. We did not have an opportunity to obtain more PET parameters such as metabolic tumor volume (MTV) and total lesion glyclolysis (TLG) to compare in IDC-DCIS and pure IDC cases. Significantly increased Tc-99m V-DMSA uptake was reported in patients with IDC-DCIS as compared to pure IDC (13). In their study, any focally increased Tc-99m V-DMSA accumulation was regarded as associated with invasive pathology, while any other pattern of more widespread diffuse uptake was considered as corresponding to pre-invasive lesions (CIS, epithelial hyperplasia). In our study, axillary lymph node metastases were more common in pure IDC than IDC-DCIS cases per histopathology. Per PET, axillary lymph node metastases were slightly higher in pure IDC than IDC-DCIS (61.5% versus 56.8%). PET was false negative for axillary metastases in 5 cases in each group. This was mainly due to early metastases with small size tumor which was below PET resolution and detected via SLN biopsy. In our recent study, combined evaluation of FDG PET/CT and single photon emission computed tomography/CT (SPECT/CT) SLN images allowed better assessing FDG uptake particularly in the SLN (27). PET was false positive in 3 cases with IDC-DCIS, which could be due to inflammatory uptake of FDG in axillary lymph nodes or result of false negative SLN biopsy (28). Abnormal FDG uptake in distant tissues (consistent/suggestive or suspicious/ questionable for distant metastases) was higher in IDC-DCIS than pure DCIS cases in our study. However, given relatively small number of our pure IDC cases and also lack of histopathological proof of distant metastases, the significance of this finding is uncertain. 

 In our study, multifocal uptake and multifocal tumor was more common in patients with coexisting IDC-DCIS pure IDC cases. Our preliminary findings may support the idea that DCIS may be the precursor for IDC in IDC-DCIS cases and also raise a question if there may be radiologically/pathologically undetected multifocal tumor in IDC-DCIS cases with unifocal tumor. Rath et al. conducted a study in patients who had palpable, invasive carcinomas of the breast, and had undergone a primary breast-conserving therapy and found that patients who had multifocal disease, accompanying DCIS, involvement of regional lymph nodes, high-grade breast cancer, lympho-vascular invasion or negative hormone-receptor status, were significantly more likely to have undergone incomplete removal of tumor tissue and these patients thus required a secondary surgery (29). 

 As the histopathology result only showed the presence of DCIS but not the exact location and size of it, we cannot determine if the multifocal uptake on PET was due to multifocal tumor and/or DCIS. Given higher avidity of FDG in IDC than DCIS, areas of high uptake may favor multifocal tumor and low uptake may be from small foci of tumor versus DCIS. Multifocal uptake in the breast usually indicates multifocal/ multicentric tumor. However, focal uptake in breast could also be from coexisting-DCIS if not smaller than 1-2 cm, intramammary nodal metastasis, and benign breast lesions such as fat necrosis, fibroadenoma, atypical ductal hyperplasia, intraductal papilloma, focal hyperplasia, infection or inflammation (30). 

 Assessing the whole breast without significant interference from the background breast tissue is an advantage of PET over other radiological imaging modalities to detect multifocal tumor. Normal breast FDG uptake is usually low and does not obscure the visualization of hypermetabolic foci. However, PET cannot detect microscopic foci and foci smaller than 6-8 mm. Diffusely increased breast uptake is not very common but if present it may obscure the visualization of small hypermetabolic foci. Bilateral diffuse breast uptake could be physiological (age related, due to dense breasts or related to menstrual cycle phases) or due to pregnancy/lactation (30, 31). Unilateral diffuse breast uptake could be due to mastitis, benign hyperplasia, inflammatory carcinomas, or diffuse lymphomatous involvement (30, 31). Mastitis may also show heterogeneous distribution of activity and appearance of multifocal uptake.

 One of the limitations of our study was the small number of pure IDC cases which might affect statistical results when comparing various parameters in IDC-DCIS and pure IDC cases. As the majority of IDC patients have coexisting DCIS, we had only small number of pure IDC patients. Studies in larger number of patients may provide more accurate results. The other limitation of our study was partial surgery to be performed in some of our patients. Although this was not a major problem for IDC-DCIS patients, it may cause false negative DCIS result in pure IDC group. However, in pure IDC group, partial surgery was done only in 4 of 13 patients and in other 9 patients with mastectomy whole breast was examined pathologically. Although our study has some limitations, it is the only study reporting/comparing FDG PET findings in IDC-DCIS and pure IDC cases.

## Conclusion

 In our preliminary findings, multifocal breast FDG uptake and multifocal tumor appears to be more common in patients with IDC-DCIS than pure IDC cases. There is no significant difference in SUV and size of the primary tumor in IDC-DCIS and pure IDC cases. Axillary lymph node metastases appear to be more common in pure IDC cases than IDC-DCIS.

## Disclosure

 No potential conflict of interest relevant to this article was reported.
